# Can the Brain Benefits of Exercise Be Enhanced Without Additional Exercise?

**DOI:** 10.29245/2572.942x/2016/2.1027

**Published:** 2016

**Authors:** J. Leigh Leasure, Rebecca West

**Affiliations:** 1 Department of Psychology, University of Houston, 126 Heyne Building, Houston, TX 77204-5022, United States; 2 Department of Biology & Biochemistry, 3455 Cullen Boulevard, Room 342, Houston, TX 77204-5001, United States

**Keywords:** Exercise, Physical Activity, Neurogenesis, Temperature, Hippocampus, Brain Disease

Exercise is increasingly becoming accepted as “medicine” for diseases of both brain and body^1^. For the brain, exercise offers chemical, cellular and structural benefits, including enhanced generation of new neurons, glia and blood vessels^2-5^, increased expression of neurotrophins (such as brain-derived neurotrophic factor (BDNF)^6,7^), dendritic remodeling^8,9^ and stabilization of stress responses^10^ and inflammatory signaling^11^. These mechanisms of action directly counteract those present in disease states. For example, the depressed brain is characterized by decreased synaptic plasticity, hippocampal neurogenesis and BDNF^12^, all of which can be reversed by exercise.

## Why is it Important to Study How Brain Exercise Benefits can be Enhanced?

While a great deal is known about how exercise benefits the brain, there are several reasons why research is needed on how to reap those benefits with minimal exercise time. First, most people do not exercise much. Research-based guidelines for weekly physical activity for various age groups have been proffered by many public health agencies, including the World Health Organization^13^. While these help to raise global awareness of the importance of exercise for the maintenance of health, most people do not meet minimum guidelines. For example, among Americans, only about 20% of adults, and 27% of adolescents meet the minimum exercise recommendation for their respective age groups^14^. One of the most commonly stated barriers to physical activity is a lack of time^15^, prompting studies of high-intensity, short duration exercise regimens, which may offer benefits similar to those of much longer duration^16^. High intensity exercise may work for healthy people, but another barrier to exercise is physical disability. For example, deconditioning and paresis often occur in disorders such as stroke, in which the chemical and cellular effects of exercise would be of great benefit to the brain, yet the body cannot sustain much physical activity^17^. Because of situations in which brains that need exercise are paired with bodies that cannot sustain it, the pharmacomimetics of exercise have become of great interest in recent years, given the potential utility of replicating brain exercise benefits by administering pharmacological treatments in lieu of actual exercise^18^. For some conditions, such as motor impairments due to traumatic brain injury (TBI), no effective drug treatments currently exist, making physical rehabilitation the only option^19^. Therefore, discovering ways to enhance the effectiveness of exercise would be of great benefit for motor intervention strategies for TBI. Finally, there is a delayed onset of many brain exercise benefits, such as increased neurogenesis, for which 2 weeks of exercise is necessary, or enhanced synaptic efficacy, for which 8 weeks is necessary^20^. This is an important consideration for exercise-based treatments for brain injury, as it would be advantageous to time exercise-driven neuroplasticity to occur within therapeutic windows of opportunity following injury. Thus, there are multiple situations in which it would be advantageous to increase the brain benefits of exercise, without increasing exercise time.

## The Volume and Type of Exercise Influence its Neural Benefits

Brain exercise benefits translate into cognitive improvements, particularly in populations that experience cognitive decline, such as the aged^21,22^. Much attention has been given to studying what volume (total amount, taking into account intensity, frequency, duration and longevity of exertion) of exercise yields maximum cognitive effects. While some studies indicate that there are cognitive benefits incurred by acute bouts of exercise in both children^23^ and adults^24^, an extensive literature indicates that these effects are not long-lasting. In other words, exercise must occur long term in order for brain benefits to be maintained^25-27^. Indeed, a recent study found a positive association between lifelong leisure time physical activity and cognitive function in late middle age^28^. In aged adults, adding 2-3 moderate walking or resistance training sessions a week can prevent cognitive decline and promote memory^29,30^, suggesting that exercise need not be daily or strenuous in order to confer neural benefits.

In addition to studies on the optimal volume of exercise, the type of exercise that produces maximum cognitive benefit has also received much attention. Some studies indicate that repeated, short bursts of high intensity exercise may provide more cognitive benefits^31^ and increased learning, BDNF and catecholamine levels^32^ in comparison with lower-intensity sustained exercise^26^. However, other results suggest that the overall level of daily physical activity is most important for brain health, regardless of whether it results from an active lifestyle or from a structured exercise regimen^33^.

## Changing the Environment in which Exercise Occurs Changes the Way it Impacts the Brain

Much attention has thus been directed at studying aspects of exercise that influence its brain health benefits. A different approach is to investigate environmental influences, such as whether the exercise occurs in a social setting. Exercise has been shown to stimulate hippocampal neurogenesis more effectively in socially-housed rats, compared to those that lived in isolation^34,35^. In humans, it has been shown that cognitive activation during exercise, such as the cognitive demands resulting from team sports, may enhance the brain benefits^36^. The influence of social interaction on exercise-driven neurogenesis raises the question of what other aspects of the environment can influence brain exercise benefits.

One possibility is ambient temperature, which has been shown to regulate cell proliferation in the reptile brain^37^, and which has marked effects on exercise in both humans and animals. Exercise is a significant physiological challenge to the brain because it produces heat^38^, and brain function is optimal only within a narrow temperature range. Regulatory mechanisms balance heat production and loss^38,39^ and prevent the brain from being damaged by an exercise-induced increase in core temperature^40,41^. Exercise at hot ambient temperature challenges these regulatory mechanisms^40,42^, and decreases time to exhaustion^43^. Exercise in the cold, however, improves physical performance^43^ and possibly even neural function^44^. Both humans^43^ and rats^45^ reach volitional fatigue later when exercising in cold ambient temperature, compared to hot. A recent study found that brain temperature rose when rats exercised at a neutral temperature (25°C), but not when they ran in the cold (12°C). Thus, prevention of exercise-induced increases in brain temperature may contribute to the increased exercise time and enhanced performance possible in cold ambient conditions^45^.

## Ambient Temperature Influences Brain Exercise Benefits

Taken together, these data raise the question of whether exercise would be even more beneficial for the brain if performed in the cold, as this would prevent the increase in brain temperature that normally occurs during exercise at room or hot temperatures. We recently addressed this question by examining the effects ofexercise at different ambient temperatures on hippocampal neurogenesis^46^. Of the many neural benefits of exercise, enhanced neurogenesis is of particular interest because it is associated with enhanced cognition^47,48^, whereas decreased neurogenesis (which occurs, for example, due to brain aging or in the case of radiation therapy for brain cancer) has been linked to cognitive impairment^49-51^. We recently compared exercise-induced hippocampal neurogenesis in rats that exercised in cold (4.5° C), hot (37.5°C) or neutral (20°C) ambient temperature. We predicted that exercise in the cold (but not the hot) condition would increase the number of newly generated neurons in the hippocampal dentate gyrus (DG) above and beyond exercise at room temperature.

We compared the number of newly generated neurons (assessed using doublecortin immunohistochemistry) in the hippocampal DG between groups that exercised or remained sedentary in the three temperature conditions. Our study yielded several interesting findings. First, temperature alone did not affect the number of newly generated neurons. In other words, sitting in the cold or the heat had no effect on hippocampal neurogenesis. Second, animals exercising in cold or hot conditions ran a much shorter distance and spent less time running, compared to animals that exercised at room temperature. Surprisingly, although they ran much less, animals that exercised in the cold or hot conditions had the most newly generated neurons in the hippocampal DG. Thus, with less total exercise distance and less total exercise time, more new neurons were generated, suggesting that manipulating the temperature at which exercise occurs may be a straightforward way to maximize exercise-driven neurogenesis.

## Future Directions

The fact that exercising in the heat resulted in more new neurons than exercise at room temperature was counter to our hypothesis. In terms of increasing brain exercise benefits, it is hard to imagine people embracing the idea of exercising in the heat (although the popularity of hot yoga classes suggests otherwise). Therefore, to follow up on our initial findings, we plan to focus on the effects of cold ambient temperature on brain exercise benefits. The mechanisms underlying the effect are presently not known, but there are many possibilities. Compared to exercise at room temperature, exercise in the cold may more effectively decrease circulating stress hormones or inflammatory factors, and/or increase trophic factors such as BDNF, all of which would be expected to enhance the effect of exercise on neurogenesis. It is also possible that exercise in the cold stimulates quiescent neural stems cells to divide. Also unknown is whether there are additional brain benefits of running in the cold beyond the effects on neurogenesis. For example, it would be useful to examine the effects of running in the cold on glia and vasculature, as a thorough characterization would clarify what brain disease states could best be treated by exercising in the cold. Finally, it will be important to determine whether exercising in the cold enhances cognition. Exercise in general improves cognition^21,22^, but it will be interesting to see whether the same effects can be achieved with a limited amount of exercise performed at cold ambient temperature.

To summarize, although exercise provides a wealth of brain benefits, it is important to investigate ways in which to maximize them, without additional exercise. It is unfortunate that brains in need of the restorative benefits of exercise are frequently paired with bodies incapable of sustained physical activity. Exercising in the cold may be one way in which to achieve maximal exercise-driven neuroplasticity, with minimal exercise effort.
